# Supplementary material for: Single-cell analysis of a high-grade serous ovarian cancer cell line reveals transcriptomic changes and cell subpopulations sensitive to epigenetic combination treatment
Source: PLoS One. 2022 Aug 3;17(8):e0271584. doi: 10.1371/journal.pone.0271584 (PMC9348737; doi:10.1371/journal.pone.0271584)
Supplement: S1 Fig — A and B) OVCAR3 cells were treated with DMSO, 5 μM. EZH2i, 50 μM RAC1i or combination for 48 hours. cDNA was made from RNA collected from bulk populations of cells and RT-qPCR was performed. Expression of the indicated gene was normalized to a housekeeping gene and then to the untreated control. N = 3. Graphs depict mean +/- SEM. *P<0.05, **P<0.01, ***P<0.001. (DOCX) [file pone.0271584.s001.docx]

**
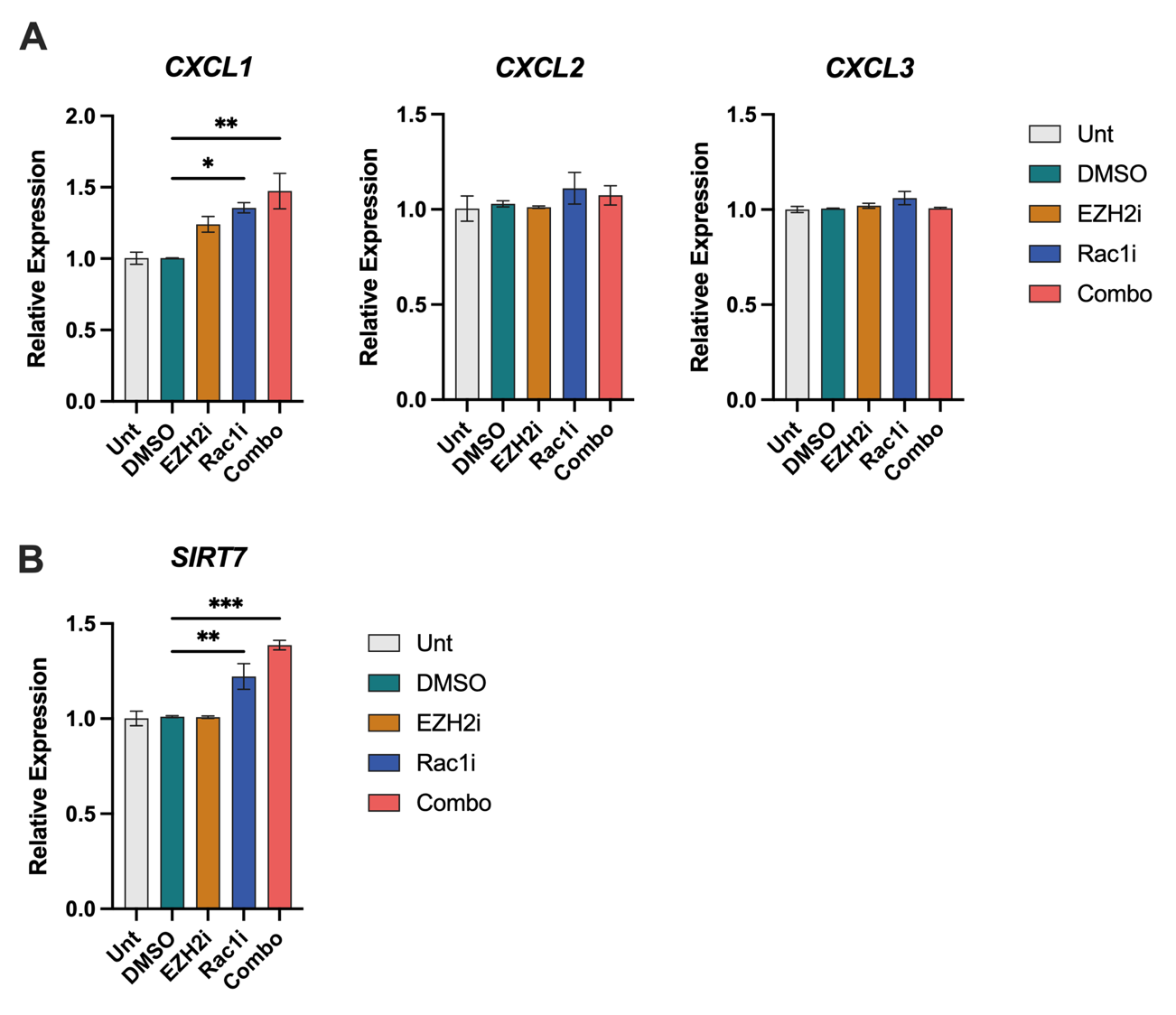
**

**S1 Fig.** **RAC1 inhibition induces altered expression of some genes in bulk OVCAR3 cells.** A and B) OVCAR3 cells were treated with DMSO, 5 μM

EZH2i, 50 μM RAC1i or combination for 48 hours. cDNA was made from RNA collected from bulk populations of cells and RT-qPCR was performed. Expression of the indicated gene was normalized to a housekeeping gene and then to the untreated control. N=3. Graphs depict mean +/- SEM. **P*<0.05, ***P*<0.01, ****P*<0.001.
