# Supplementary material for: Single-cell analysis of a high-grade serous ovarian cancer cell line reveals transcriptomic changes and cell subpopulations sensitive to epigenetic combination treatment
Source: PLoS One. 2022 Aug 3;17(8):e0271584. doi: 10.1371/journal.pone.0271584 (PMC9348737; doi:10.1371/journal.pone.0271584)
Supplement: S2 Fig — Violin plots of expression levels of indicated genes in the different clusters and sample types. (DOCX) [file pone.0271584.s002.docx]

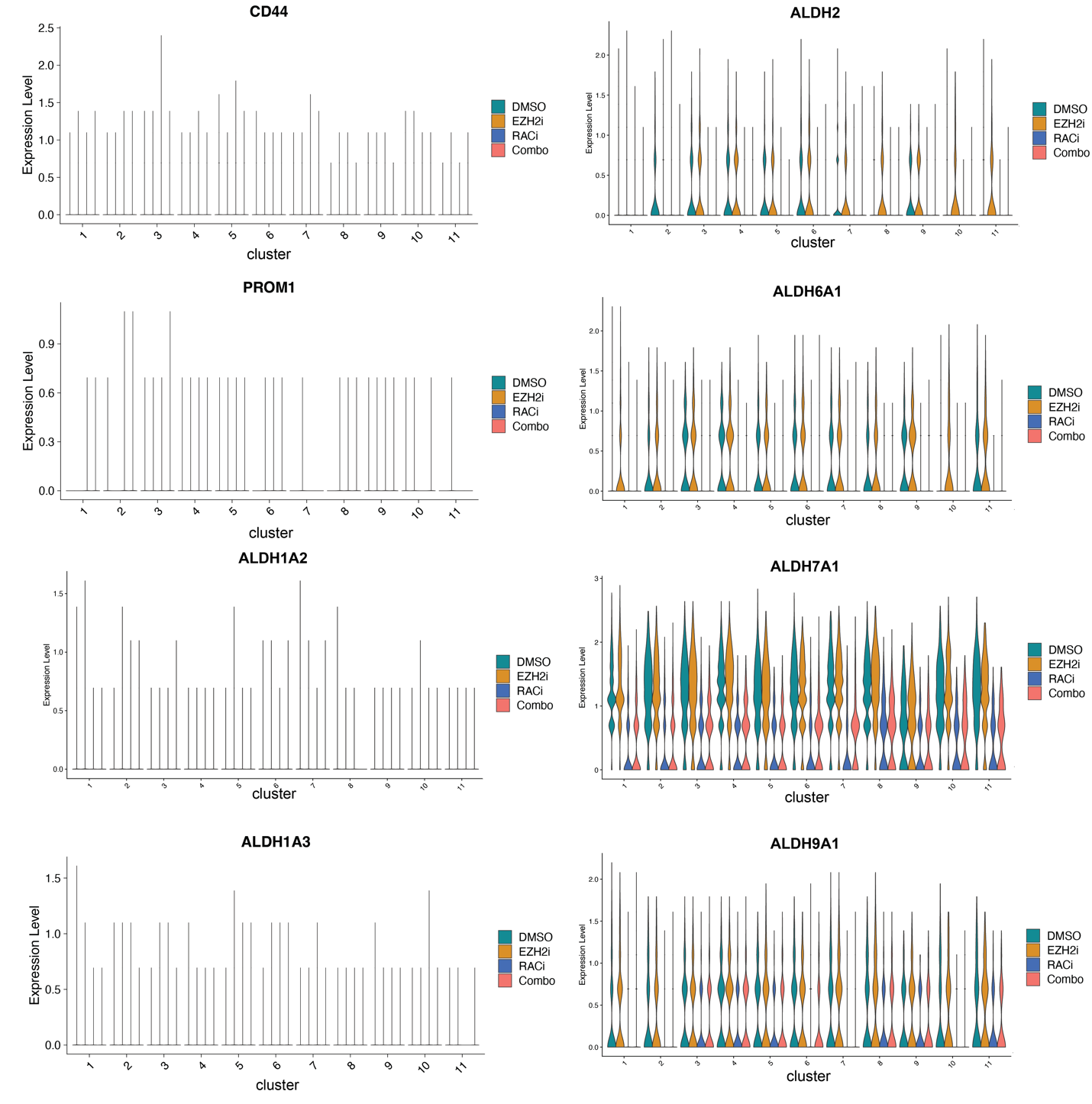


**S2 Fig.** **RAC1 inhibition reduces expression of several ALDH isoforms in most clusters.** Violin plots of expression levels of indicated genes in the different clusters and sample types.
