# Supplementary material for: Single-cell analysis of a high-grade serous ovarian cancer cell line reveals transcriptomic changes and cell subpopulations sensitive to epigenetic combination treatment
Source: PLoS One. 2022 Aug 3;17(8):e0271584. doi: 10.1371/journal.pone.0271584 (PMC9348737; doi:10.1371/journal.pone.0271584)
Supplement: S1 Table — (DOCX) [file pone.0271584.s003.docx]

| Cluster | Top genes enriched in each cluster |
| --- | --- |
| 1 | SNHG8, SNHG17, SNHG12 |
| 2 | MT2A, MT1X, GADD45B, NFKBIA, HES1, ID2, JUNB, ID1, MT1F, EID2 |
| 3 | SLPI, TM4SF1, CLU, NMU, MRPS6, UPK1B, GPRC5A, LRPAP1 |
| 4 | HIST1H4C, HIST2H2AC, HELLS, HIST1H1E, MKI67, PCLAF, TYMS, TUBB, RRM2, TOP2A |
| 5 | CXCL1, CXCL8, UCA1, CA2, IER3, CXCL2, FLG, IL6, CD70, CLDN1 |
| 6 | MALAT1, NEAT1, CP, SCD, KCNQ1OT1, MT-CO2, CDH6, MT-CO3, MUC1, MT-CO1 |
| 7 | PLCG2, FLG, AL627171.2, KCNQ1OT1, MT- ND6, MTRNR2L12, HEXIM1, MT-ND5, AC108134.2, AL355075.4 |
| 8 | CCNB1, CDKN3, HMMR, PTTG1, AURKA, CCNB2, DEPDC1, UBE2S, DLGAP5, CDC20 |
| 9 | GPX3, FTL, HIST1H4C, FTH1, DHCR24, SYNGR2, NME4, MYBL2, CCDC85B, GUK1 |
| 10 | CRYAB, FHL2, KRT7, S100A11, LGALS3, LGALS1, PPP1R14A |
| 11 | SNHG19, AC010997.5, AC103591.3, SNHG3, HOXA-AS2, SNHG4, IGFL2-AS1, SNHG25, CSKMT, AC245014.3 |

**Supplementary Table S1** – Top genes enriched in each cluster
