# Supplementary material for: Single-cell analysis of a high-grade serous ovarian cancer cell line reveals transcriptomic changes and cell subpopulations sensitive to epigenetic combination treatment
Source: PLoS One. 2022 Aug 3;17(8):e0271584. doi: 10.1371/journal.pone.0271584 (PMC9348737; doi:10.1371/journal.pone.0271584)
Supplement: S3 Table — (DOCX) [file pone.0271584.s005.docx]

| **Cluster** | **DMSO** | **EZH2i** | **RAC1i** | **Combo** |  |  |
| --- | --- | --- | --- | --- | --- | --- |
| 1 | 1314 | 1213 | 1850 | 1362 |  |  |
| 2 | 518 | 568 | 2360 | 2021 |  |  |
| 3 | 823 | 907 | 1332 | 1132 |  |  |
| 4 | 993 | 1102 | 1070 | 841 |  |  |
| 5 | 691 | 742 | 982 | 859 |  |  |
| 6 | 953 | 849 | 684 | 580 |  |  |
| 7 | 640 | 719 | 577 | 556 |  |  |
| 8 | 551 | 527 | 376 | 303 |  |  |
| 9 | 386 | 473 | 564 | 210 |  |  |
| 10 | 402 | 294 | 224 | 211 |  |  |
| 11 | 259 | 257 | 188 | 157 |  |  |
| total | 7530 | 7651 | 10207 | 8232 |  |  |
|  |  |  |  |  |  |  |

**Supplementary Table S3:** Number of cells in each cluster for each treatment group.
